# Polycystic ovary rat model exposure to 150 kHz intermediate frequency: hypothalamic-pituitary-ovarian axis at the receptor, cellular, tissue, and hormone levels

**DOI:** 10.1186/s13048-021-00914-w

**Published:** 2021-12-11

**Authors:** Stephanie Mohammed, Venkatesan Sundaram, Chalapathi R. Adidam Venkata, Nikolay Zyuzikov

**Affiliations:** 1 Department of Physics, Faculty of Science and Technology, The University of the West Indies, St. Augustine, West Indies Trinidad and Tobago; 2 Department of Basic Veterinary Sciences, School of Veterinary Medicine, Faculty of Medical Sciences, The University of the West Indies, St. Augustine, West Indies Trinidad and Tobago; 3 Department of Clinical Medical Sciences, Faculty of Medical Sciences, The University of the West Indies, St. Augustine, West Indies Trinidad and Tobago

**Keywords:** Electromagnetic radiation, Polycystic ovary, Estradiol Valerate; HPO

## Abstract

**Introduction:**

The hypothalamic-pituitary-ovarian (HPO) axis is the principal regulator of the reproductive system. The neurons in the arcuate nucleus of the hypothalamus signal the basophilic cells of the anterior pituitary to release luteinizing hormone (LH) and follicle stimulating hormone (FSH), which bind to the granulosa and theca cells of a follicle in the ovary to promote healthy follicular development. Disruption of this process at any time can lead to polycystic ovaries and, if left untreated, can lead to Polycystic Ovarian Syndrome (PCOS), one of the leading causes of infertility. A novel treatment option using 150 kHz Intermediate Frequency (IF) Electromagnetic Radiation (EMR) has been proposed to monitor the effect of this frequency during cystic development.

**Methods:**

To prove this, an experiment was conducted to study the effect of whole-body exposure to 150 kHz EMR for 8 weeks at receptor, cellular, tissue and hormonal levels on the HPO axis of 25 young cyclic female rats.

**Results:**

The results showed that 150 kHz EMR did not affect the histoarchitecture of neurons of arcuate nucleus of the hypothalamus of PCO-induced rats. It was also found that the number of basophilic cells of the pituitary gland was increased and the immunoreactivity of LH and FSH secretion increased. This EMR field also decreased the development of follicular cysts in the ovary and possibly increased the immunoreactivity of the LH and FSH receptors as well on the theca and granulosa cells of follicles in the ovary.

**Conclusion:**

There are still many limitations to this study. If properly evaluated, the results of this experiment could help develop a new non-invasive treatment option for women with PCOS in the near future.

## Introduction

The hypothalamic-pituitary-ovarian (HPO) axis is a meticulously synchronized and tightly regulated network ultimately responsible for reproductive function. Hypothalamic control of reproduction is coordinated by the pulsatile release of gonadotropin-releasing hormone (GnRH) from the arcuate nucleus of the hypothalamus through the pituitary portal system, where it acts on receptors on the surface of the anterior pituitary lobe. Binding of GnRH to their receptors triggers the synthesis and secretion of luteinizing hormone (LH) and follicle stimulating hormone (FSH) from the basophilic cells of the pituitary gland. The release of LH and FSH into the blood activates a series of events, culminating in binding these hormones to receptors in the ovary. Follicle stimulating hormone binds to receptors in the granulosa cells and LH binds to receptors in the theca cells of the ovary. These hormones act in the gonads to stimulate the production of gametes and promote the gonadal release of sex steroids, namely testosterone (T), estradiol, and progesterone (P4). In addition to controlling reproductive function in the tissues, these gonadal steroids may also modulate upstream HPO components. Each level of the HPO axis is tightly regulated but can be modulated to affect reproductive status [1].

Polycystic ovary syndrome (PCOS) is a common reproductive-endocrine disorder affecting 2–20% of women of reproductive age worldwide [2], characterized by insulin resistance, androgen excess, and ovarian dysfunction [3, 4]. Studies have highlighted the role of the HPO axis in the endocrine dysfunction of PCOS such as abnormal GnRH pulse frequency, increased LH/FSH ratio, adrenal, and ovarian androgen excess [3]. Current treatment options for PCOS mainly focus on controlling signs, symptoms and preventing complications rather than treating the condition. Therefore, finding more effective, affordable treatment options is an important issue in the treatment of PCOS. Testosterone in women is usually produced as a precursor to estradiol (E2) in the ovarian thecal cells. Serum testosterone levels are typically maintained at lower levels in women of reproductive age, because testosterone is converted to E2 by aromatase (Cyp19) enzyme in follicular granulosa cells. Serum sex hormone levels in women are tightly regulated by the HPO axis, which is associated with follicular growth in the ovary [1]. Although the molecular mechanisms are unclear, PCOS is considered a dysfunction of the HPO axis. Correction of the HPO axis dysfunction may be a potential treatment option to reverse the PCOS condition.

The effects of Electromagnetic Radiation (EMR) on the reproductive system range from dangerous, neutral, or beneficial [5]. The results of reproductive studies confirming the beneficial effects of electromagnetic waves, such as invasive procedures with laparoscopy or non-invasive procedures with Tumor Treating Fields (TTFs fields) [6–8], give hope for the application of these inventions in the treatment of PCOS in humans. The intermediate frequency (IF) range (100 to 300 kHz) of EMR (TTFs) has been successfully used to treat various cancers along with chemotherapy. Tumor Treating Field is an innovative and non-invasive cancer therapy that interrupts mitosis and selectively kills rapidly dividing cancer cells. The treatment involves the continuous delivery (over 18 h per day) of low-intensity, medium frequency (100 kHz – 300 kHz) alternating electric fields to the tumour site [9]. The optimal frequency for antimitotic effect varies by cancer type and can be adjusted for maximum anti-cancer effect. Tumor Treating Fields were found to be very effective in the treatment of ovarian cancer in a preclinical setting at 200 kHz [10]. Our laboratory demonstrated beneficial effects in an Estradiol Valverate (EV)-induced PCO rat model exposed to whole-body irradiation at 150 kHz, including an improved reproductive cycle, a reversal to the usual morphology of developing follicles, an increased number of typical developing follicles, and a reduction in the average number and diameter of follicular cysts [11]. Such reports sparked interest in discovering the effect of this frequency on the HPO axis in polycystic ovaries with the idea of exploring the option of an alternative therapy for PCO management. In addition, the effect of this frequency on HPO axis will also help to assess the safety of this mid-frequency field in non-cancerous cells. This study aims to examine, how 150 kHz electromagnetic radiation affects the HPO axis in rats with Estradiaol Valverate-induced PCO.

## Materials and methods

### Animals and experimental design

Twenty-five (25) healthy young adult Sprague Dawley (SD) female rats (12–15 weeks old) weighing 200-300 g were used in the current study. The animals were obtained from the Lab Animal Facility at the School of Veterinary Medicine, The University of the West Indies, St. Augustine, Trinidad, and Tobago. Animals were housed in polypropylene cages (3 animals per cage) measuring 40 × 24 × 14 cm in a designated experimental room at the School of Veterinary Medicine, at an ambient temperature of 22 ± 3 °C and 50–60% relative humidity, with a 12-h light/dark artificial light cycle. Standard pellet food and water were given ad libitum. All animals were acclimatized to laboratory conditions for 7 days before experiments. All animal experiments were conducted in strict compliance with the National Institutes of the Guide for the Care and Use of Laboratory Animals, and approved by the Campus Research Ethics Committee, The University of the West Indies, St. Augustine, Trinidad, and Tobago (No. CEC 310/09/17).

Animals were randomly divided into 4 groups: control (c), Estradiol Valerate (EV), EMR, and EV + EMR, such that there were 7 animals in each group, except for the control group, which consisted of 4 animals. The lower number of control was used to minimise the number of animals used for the experiments according to the ethical guidelines. In addition, we have the full number of positive controls which was sufficient for comparison. Both the EMR and EV + EMR groups were exposed to whole-body EMR at 150 kHz for eight consecutive weeks (with the exception of approximately 1 h per week required for cage changing [11, 12]. In the EV and EV + EMR groups, polycystic ovaries (PCO) were induced in the rats. The animals were administered commercial EV tablets at a single oral dosage of 4 mg per animal on the first day of the experiment [13]. The oral dosage was used versus the intramuscular dosage to observe the development of PCO rather than PCOS [13, 14]. The control and EV control groups were maintained under similar conditions without EMR.

### EMR generating system

**Fig. 1:**
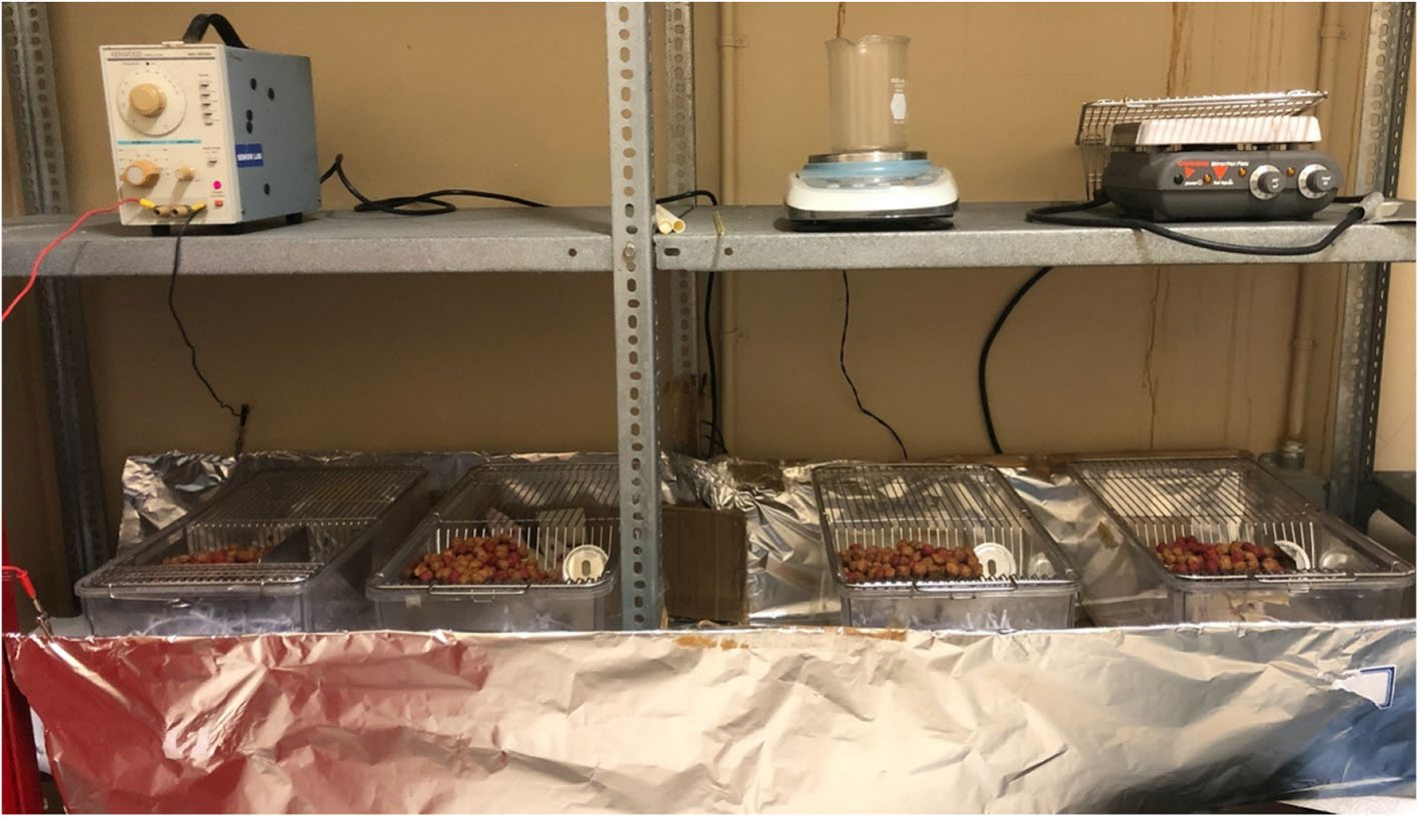
Experiment Set-up showing EMR source used to create an electric field for the experimental group

The experimental animals were placed in a uniform electromagnetic field with a frequency of 150 kHz and an amplitude voltage of 12 V. The system replicated the previous study at this frequency [11]. The EMR signal was generated by a Kenwood AG-203A oscillator (10 Hz-1 MHz) (Trio-Kenwood Electronic; Komagane, Japan) with the maximum possible result intensity. (Fig. 1: Experiment Set -up shows the EMR source that was used to generate an electric field for the experimental group) The field intensity within the experimental group was maintained at 0.3 V/cm^2^. The intensity of the EMR field in the cages was measured using the NBM-550 Broadband Field Meter (100 kHz-6 GHz) (Narda Safety Test Solutions GmbH, Pfullingen, Germany). The control and EV control groups of rats were kept in the same room as the EMR and EV + EMR groups. To avoid exposure to the 150 kHz frequency of the source, the cages containing the experimental group were surrounded with aluminum foil separately from the control groups, which were also surrounded with aluminum foil. The intensity of the EMR field was 65 ± 15 × 10^− 6^ μW/cm^2^ in the EMR + EV and EMR cages and 35 ± 15 × 10^− 9^ nW/cm^2^ in the EV and control cages. The whole room had an exposure of 0–100 nW/cm^2^. The intensity of EMR in the EMR-exposed cages was more than 1000 times higher than in the control cages.

All other sources in the room, such as light fixtures and electrical outlets, did not have the desired frequency needed for the experiment and were not considered. The geometry and positions of the cages, electrodes, and oscillator were not changed during the experiment. All details of the procedure can be verified using the appropriate reference provided.

### Assessment of estrous cycle

All animals were examined before and during the experiment by exfoliative vaginal cytology for regularity of the oestrous cycle. Only animals with three consecutive regular oestrous cycles (pro-estrous, estrous, di-estrous, met-estrous) were used for the study. Oestrous cycle was assessed by vaginal swab method. Vaginal swabs were collected daily at a constant time to reduce variability and to ensure that the evaluators were aware of inherent variations. The smears were stained with Methyl Blue and then read using an Olympus BX51 system microscope (Olympus Corporation, Tokyo, Japan). The different stages of the estrous cycle were identified using exfoliative cytology. Persistent vaginal keratinization is indicative of the development of PCO, which meant that the animals were only in the estrous phase and were not ovulating [11, 15].

### Feed intake and body weight

The animals’ feed intake and body weight were recorded on a weekly basis.

### Hormonal analysis

At the end of the exposure period, the animals were weighed and sedated intraperitoneally (i.p.) with ketamine hydrochloride at a dose of 80 mg/kg. Once the rats were sedated, they were placed under deep anesthesia by administering pentobarbital sodium at a dosage of 40 mg/kg i.p. Once the anesthesia had taken effect, 5 ml of blood was collected using a standard terminal cardiac puncture protocol. Immediately after blood collection, the animals were euthanized by an overdose with pentobarbital sodium at a dosage rate of 120 mg/kg i.p. Blood samples were centrifuged at 1500 rpm for 10 min at 4 °C to prepare serum samples, which were stored at − 80 °C. Rat ELISA kits for FSH (Catalog No. IT7322) and LH (Catalog No. IT7468) purchased from G-Biosciences, Missouri, USA, were used to measure FSH and LH serum levels in rat blood, respectively.

### Histological analysis

The brain, pituitary gland, and ovaries were dissected euthanized animals. The hypothalamus was removed by cutting the brain coronally with a razor blade at the level of the optic chiasm crainally and at the end of the crura cerebri caudally. All specimens were fixed in 10% buffered neutral formalin and processed by routine histological processing (Suverna et al., 2013). Sections were cut at a thickness of 3–5 μm using a rotary microtome *(Finesse ME, Thermo Scientific Fisher Company, Waltham, USA).* Sections were stained with haematoxylin and eosin (H&E) and analyzed using an Olympus BX51 system microscope with cellSens imaging software (version 1.12) and Olympus DP71 digital camera (Olympus Corporation, Tokyo, Japan). The hypothalamus was serially and coronally sectioned throughout and stained with H&E. Every 5th section was taken for analysis. The arcuate nucleus of the hypothalamus was carefully identified and analyzed in each section based on its anatomical position. In the ovary, all follicles were classified as either normal or atretic. Follicles with intact oocytes surrounded by layers of complete granulosa cells were considered normal. Atretic follicles, on the other hand, showed vacuolization and pyknotic nuclei within the granulosa cells, as well as occasional shrinkage of the oocytes.

### Histomorphometric analysis

Neurons in the hypothalamus were visually observed within the perimeter of the arcuate nucleus of  all animals. Cells were identified as neurons if they had a nucleus, a dendritic process, euchromatin material within the nucleus, and nuclei surrounded by cytoplasm [16, 17]. Neuronal groupings were identified and counted. Pituitary basophils were counted in 10 separate fields per section and averaged [18]. Quantitative assessment was performed by counting the number of follicles in each section of the ovary. Follicles with visible oocytes in the nuclei were counted 3 times and averaged [19]. The number of corpora lutea (CL) was also counted.

### Immunohistochemistry

Sections (5 μm thickness) of 10% buffered formalin-fixed hypothalamus, pituitary and ovary were used for immunohistochemical analysis with FSH EP257 and LH polyclonal primary antibodies. (Table 1: Antibodies used for immunohistochemical procedures). Tissues were deparaffinized and rehydrated in a descending alcohol series (100, 90, 80, 70, 30, and 0%) for 5 min each. Heat-induced epitope retrieval was performed using Vitro S. A EDTA buffer pH 8 for 20 min at 95 °C. The solution was allowed to cool at room temperature for 20 min. Blocking was performed for 10 min with peroxidase solution (Ref. MAD-O21540Q-125, Master diagnostica, Granada, Spain). Sections were incubated with the primary antibody for 10 min; immunoreactivity was visualized using a Master Polymer Plus Detection System (Ref. MAD-000237QK, Master diagnostica, Granada, Spain). Counterstaining with hematoxylin was performed and slides were analyzed. Immunohistochemistry results were semiquantitatively evaluated by adopting a Modified Allred Scoring System used to assess Hormone receptors in breast cancer by a pathologist [20]. The density of cells was scored as high (> 15), medium (10–15) and low (< 10) positive cells. The staining intensity was scored as strong (3), intermediate (2) and weak (1).

**Table 1:** Antibody used for immunohistochemistry procedure

Antibody	Species	Clone/Cat-	Method	Dilution	Source
Primary					
FSH	Rabbit	EP257	IHC	1:50–100	Vitro (master diagnostica), Granada, Spain
LH	Rabbit	Polyclonal	IHC	1:50–100	Vitro (master diagnostica), Granada, Spain
Secondary					
Labeled Polymer-HRP	Rabbit Goat	K4065	IHC		Dako Envision, CA, USA

*FSH* follicle-stimulating hormone, *LH* luteinizing hormone, *IHC* immunohistochemistry

### Statistical analysis

Data were analyzed using IBM SPSS Statistics V21 software (Armonk, New York, USA). Descriptive statistics were calculated for each group in the experiment. Normal distribution was checked and a nonparametric Kruskal-Wallis test was performed. The threshold for statistical significance was set at *p* = 0.5.

## Results

### Effect on feed intake and body weight

**Fig. 2:**
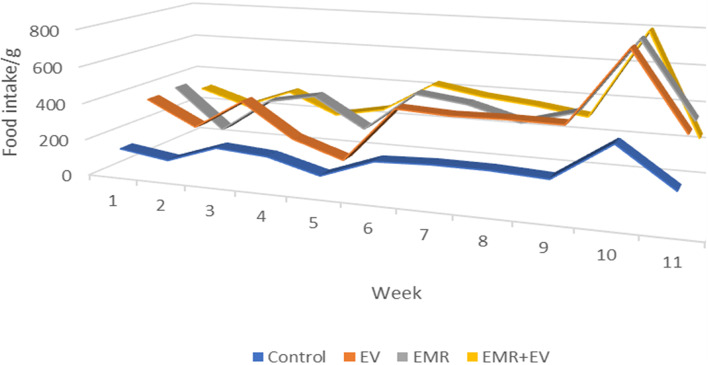
Weekly Feed intake (grams) of animals

There were no statistically significant differences in weekly feed intake or bodyweight among all groups (Fig. 2: Animals’ weekly feed intake; Table 2: Animals’ weekly body mass).

**Table 2:** Weekly Body mass (grams) of animals

	Week 0	Week 2	Week 4	Week 6	Week 8
Control	249.35 ± 10.23	252.75 ± 15.65	237.50 ± 11.15	266.50 ± 9.68	284.25 ± 12.44
Control EV	265.64 ± 44.51	274.57 ± 35.62	280.29 ± 34.49	297.00 ± 39.00	304.57 ± 38.25
EMR	290.12 ± 41.39	301.43 ± 23.54	271.29 ± 43.41	272.14 ± 27.32	301.57 ± 42.91
EMR + EV	267.43 ± 35.87	271.86 ± 33.70	248.71 ± 46.94	277.86 ± 38.37	284.57 ± 35.06

Values are expressed as Mean ± Standard Deviation of the Mean (SEM); (*n* = 7 for all groups, *n* = 4 for control)

### Effect on the histological structure of the HPO axis

#### Hypothalamus

**Fig. 3:**
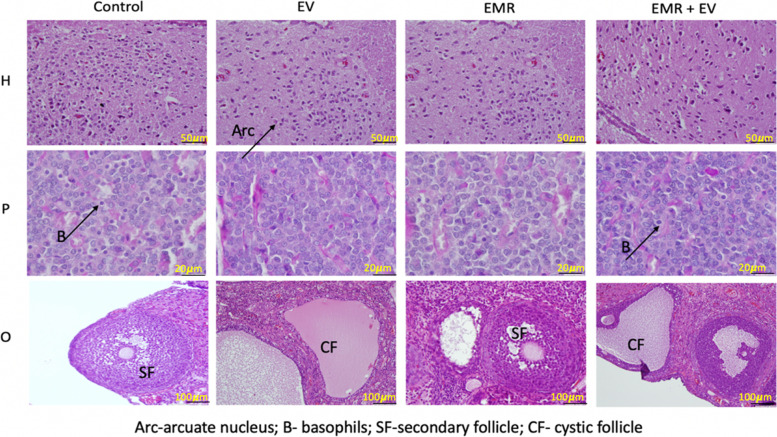
Histology of the hypothalamus, pituitary, and ovary. The photomicrograph shows neurons of the arcuate nucleus (single cluster of cells), basophil cells (poorly basophilic stained cells) of the anterior pituitary, and secondary follicle (Control-normal, EV-cystic, EMR-degenerated, EMR + EV-thin layers of granulosa, and theca cells) among each group. Arc - arcuate nucleus; B - basophil cell; SF - secondary follicle; CF – cystic follicle

The arcuate nucleus of the hypothalamus was readily identifiable in all groups studied as a single, intact large cell cluster containing a homogeneous population of relatively large neurons located at the ventral border of the hypothalamus on either side of the third ventricle (3V) near the median eminence. The large neurons were interspersed with numerous astrocytes. The nucleus contained mainly, densely stained multipolar neurons ranging from 12.5 to 17.5 μm in diameter. A few brightly stained round cells with a diameter of 7.5 μm were present in all groups examined. No changes in histoarchitecture of these neurons of the hypothalamus were observed between the groups. (Fig. 3: Histology of the hypothalamus, pituitary and ovary).

#### Pituitary gland

The parenchyma of the anterior lobe of the pituitary gland was observed with clusters and strands of cells intermingled with a large amount of connective tissue rich in sinusoidal capillaries in all groups. (Fig. 3: Histology of the hypothalamus, pituitary and ovary).

The basophilic cells appeared as clusters of different sizes and shapes in all groups, but the cells were densely packed and present in lower numbers in the EV and EMR groups. Two types of basophilic cells, regular and irregular in shaped, with dark oval nuclei with dark secretory granules in the cytoplasm were noted in all groups. The irregularly shaped cells were abundant in EMR and EV + EMR groups. The number of basophilic cells was reduced in EV group (13.19 ± 2.41). When EMR was added, the number of basophilic cells increased (EV + EMR: 21.20 ± 3.03) which was similar to the control group (27.71 ± 5.35). However, the number in the EMR control group (15.00 ± 8.69) was lower than the control group but higher than the EV group. The EV group also had increased acidophilic cells, which were smaller, round or irregular shaped and seen as strands. They were also abundant in the EMR and EV+ EMR groups. Chromophobe cells were round to regular cells found without much variation in all groups. In addition, the posterior lobe consisted of thin, nonmyelinated nerve fibers with associated glial cells overlapping with the nerve fibers. No significant differences were observed between groups in the posterior pituitary lobe.

#### Ovary

**Fig. 4:**
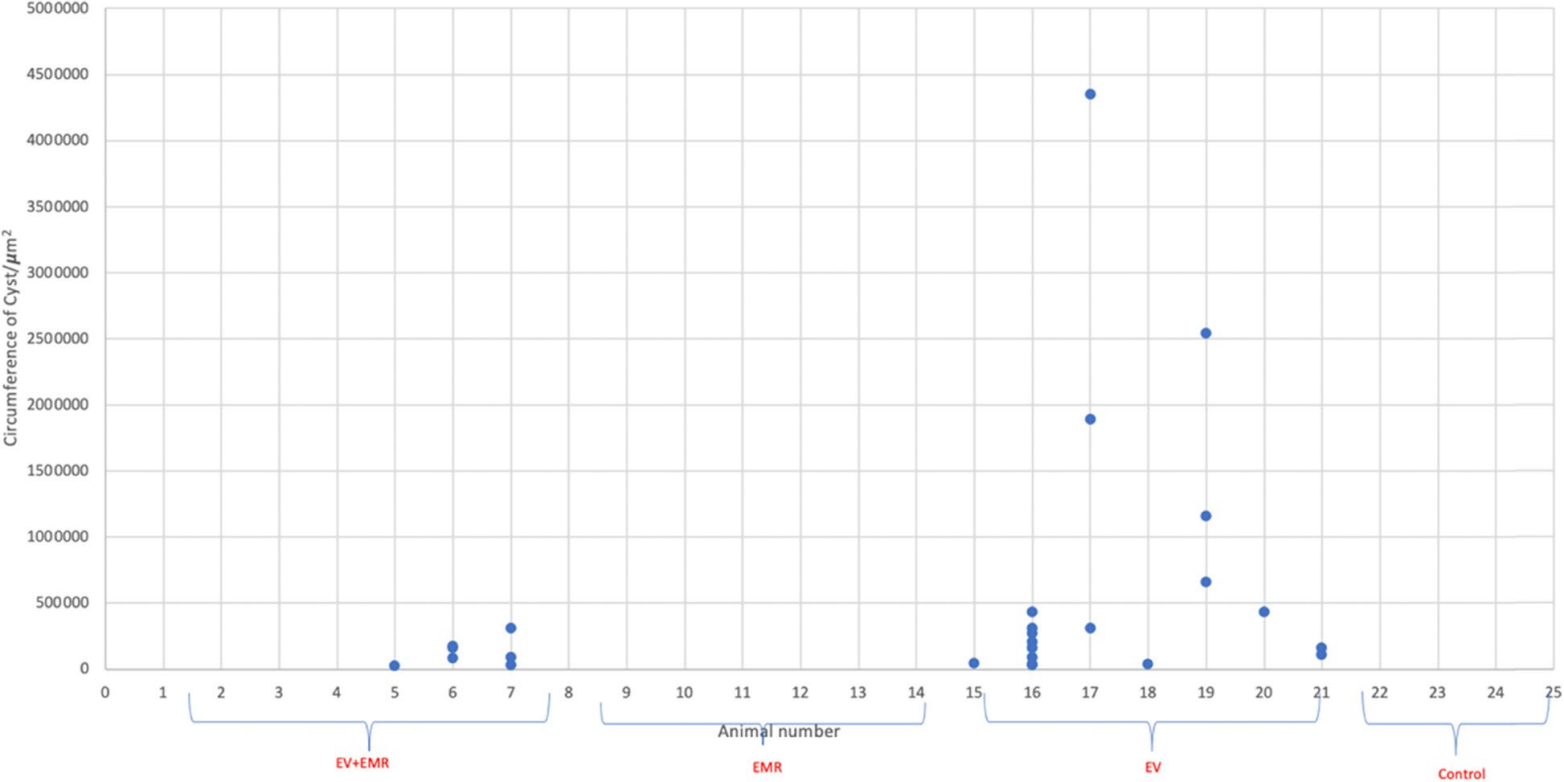
The number of Cysts and Circumference of Cysts for each group of animals. The graph shows multiple follicular cysts (EV group), a reduced number of follicular cysts (EV + EMR group), and no signs of cysts (Control and EMR control group)

**Fig. 5:**
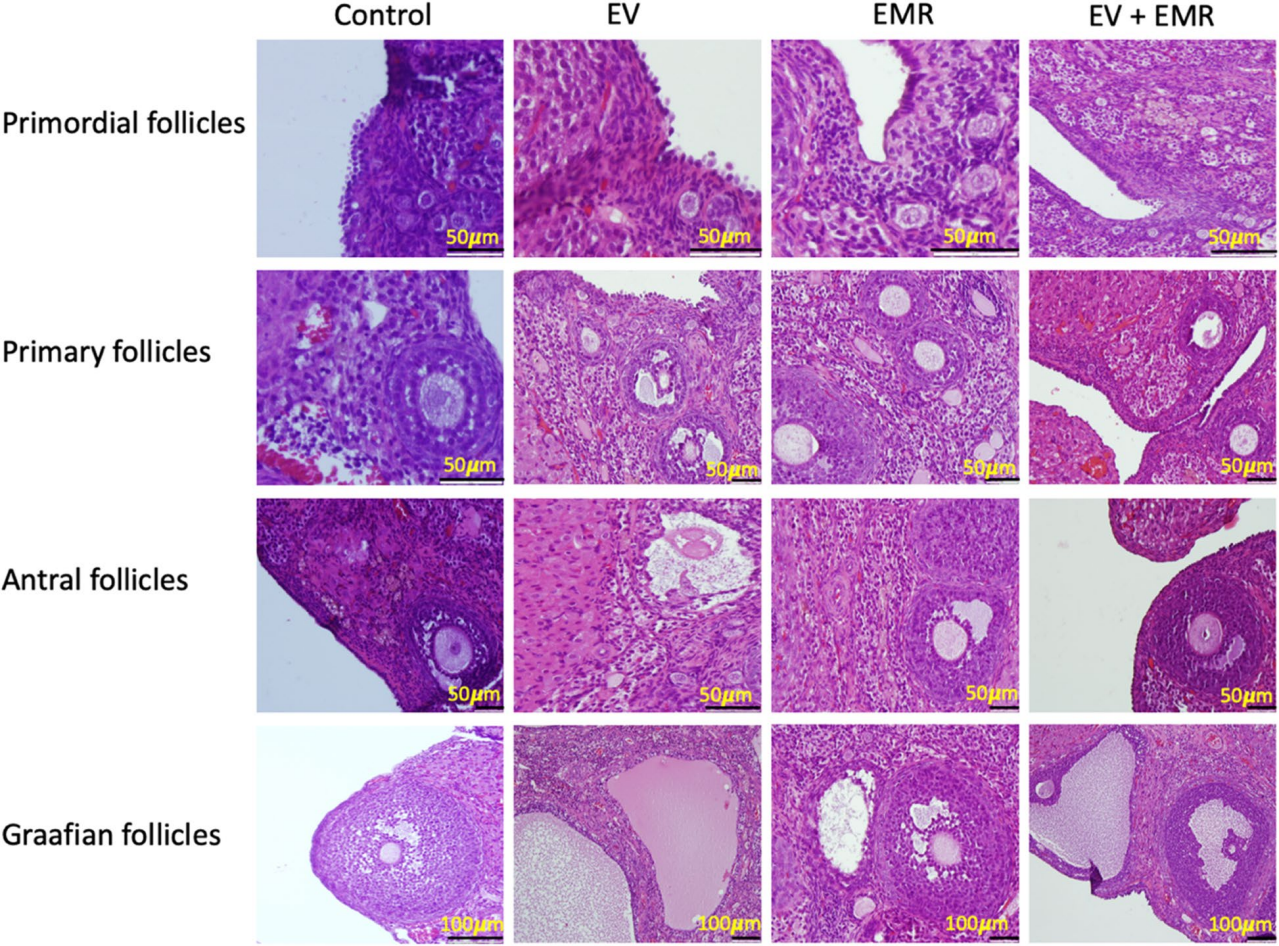
The plate showing detailed follicles among all groups. The photomicrograph shows: primordial, primary, antral, graffian, and cystic follicles among the groups. Control- has normal follicular development; EV- degenerated primary follicles, pyknosis, degeneration in the antral follicle and cystic follicle formation; EMR- atresia and signs of degeneration in the secondary follicles: EV + EMR- normal secondary and graffian follicle along with follicle appearing to go towards cystic or oocyte release

There was a significant change in the number and size of cystic follicles. (Fig. 4: The number of cysts and the size of cysts for each group of animals), which was similar to an experiment performed earlier [11]. The difference was control and EV (*p* = 0.00); EV and EV + EMR (p = 0.00); control and EV + EMR (*p* = 0.73). Reduced number and size of cysts were observed in EV + EMR group. Ovarian follicles at different stages of development were normal and intact in rats of the control group. The preantral and antral follicles showed signs of degeneration, including cell pyknosis, thin granulosa cell layer, numerous cystic follicles, thickened theca layer, distorted zona pellucida, and cumulus oophora and blood stasis, and a reduced number of CL in the EV group. The EV + EMR group showed little evidence of distortion from the antral follicle to the mature follicle. Follicles at different stages were observed in this group (almost similar to the control), with a lower number of cysts present. (Fig. 5: The plate showing detailed follicles in all groups) The EMR group showed normal follicular development with little evidence of distortion at the antral stage. Reduced number of cyst formation with thickened granulosa cells and normal theca cell layers was observed in EV + EMR group. The antral cavity of cysts in EV+ EMR group was smaller with less granulosa pyknosis compared to EV group.

### Effect of FSH and LH expression in the HPO axis

**Fig. 6:**
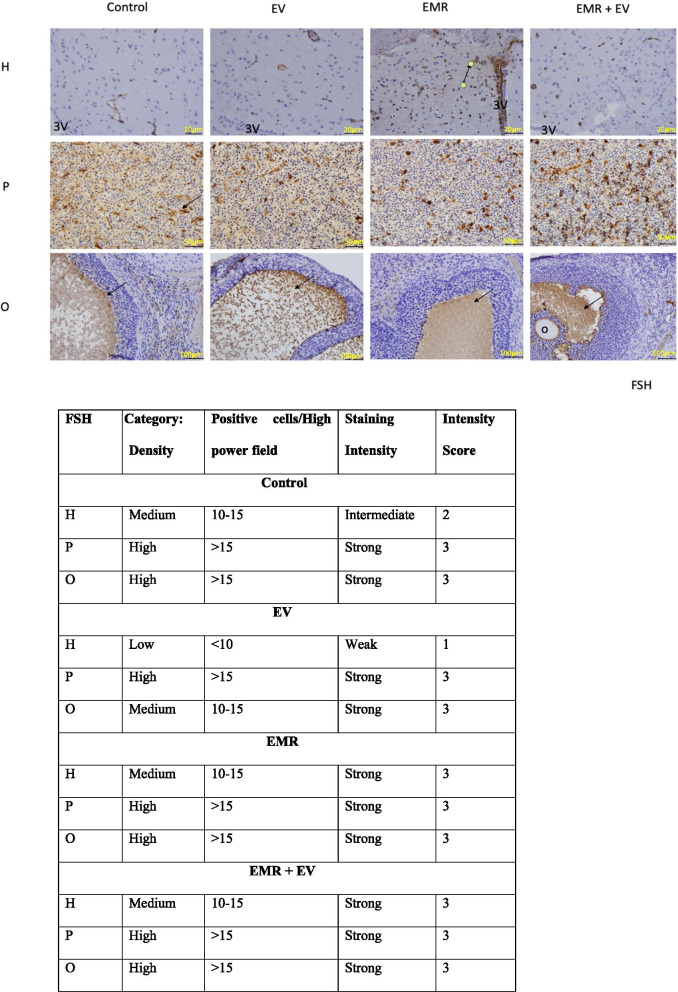
Immunohistochemical analysis of FSH reactive cells of the HPO axis. Photomicrograph showing the neurons in the arcuate nucleus around the third ventricle (3 V) in the hypothalamus (H), FSH reactive cells in the pituitary (P), and matured follicles with oocyte (o) and a comparison of the theca and granulosa cell layers among each group. Simultaneously there is a table showing qualitative analysis for FSH reactive cells (brown color with arrow pointing) among the groups in the HPO axis. Table showing modified Allred coring method

**Fig. 7:**
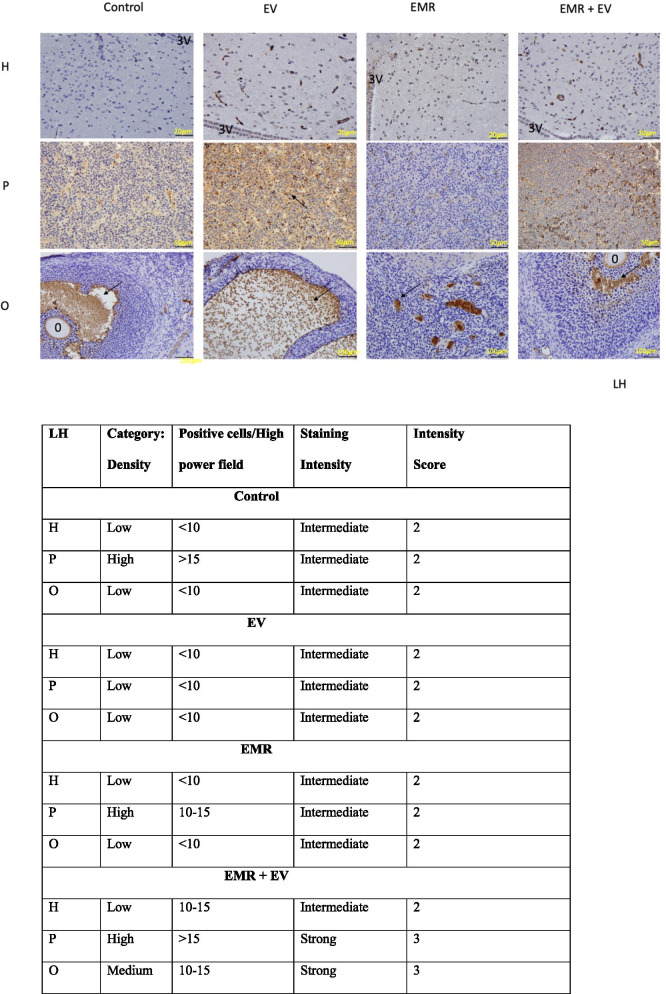
Immunohistochemical analysis of LH reactive cells of the HPO axis. Photomicrograph showing the neurons in the arcuate nucleus around the third ventricle (3 V) in the hypothalamus (H), LH reactive cells in the pituitary (P), and matured follicles with oocyte (o) and a comparison of the theca and granulosa cell layers among each group. Simultaneously there is a table showing qualitative analysis for FSH reactive cells (brown color with arrow pointing) among the groups in the HPO axis. Table showing modified Allred scoring method

(Fig. 6: Immunohistochemical analysis of FSH reactive cells of HPO axis and Fig. 7: Immunohistochemical analysis of LH reactive cells of HPO axis)

#### Hypothalamus

Immunohistochemical analysis revealed that FSH-positive and LH-positive staining was distributed in the cell bodies and around the nuclei of neurons around the 3 V in the arcuate nucleus of the hypothalamus. The EV group had a low density of FSH reactive cells (< 10) compared to the control group, which has an intermediate density of FSH cells (10–15). Both the EMR and EMR + EV groups had a medium density of FSH cells (10–15) with strong staining. All groups showed similar quantification of cells with low LH and moderate staining intensity. These results suggest that EMR may not affect the feedback of FSH and LH to the arcuate nucleus of the hypothalamus. But, GnRH-positive was not checked, which is a limitation of this result.

#### Pituitary gland

According to the FSH and LH immunohistochemistry, the FSH- and LH-positive staining was randomly distributed in the cytoplasm and membranes of the anterior pituitary tissue. All groups had high density of FSH reactive cells (> 15) with strong staining in basophilic cells. However, the EV group had a lower density of LH cells (< 10) compared to the control group which had a high density of LH reactive cells (> 15). The EMR and EMR + EV groups also had a high density of LH reactive cells (> 15). The LH reactive cells in all groups had moderate staining. These results suggest that EMR could have a neutral effect on the number of LH and FSH reactive cells. Or, from another perspective, EMR appears to allow the increase of LH reactive stains because, the EV + EMR group had higher LH densities of cells when compared to the EV group.

#### Ovary

Immunohistochemistry of FSH- and LH-positive staining was distributed around the follicles in the ovary. The density of FSH reactive cells in the EV group was lower (10–15 cells) than in the Control group, which had a high density (> 15) with strong staining. The EMR and EMR + EV group also had high a high density of FSH cells. However, LH reactive cells were low (< 10) in all groups, except the EMR + EV group which had medium density of LH cells and showed moderate staining. The results from this analysis suggest that EMR may have a positive effect in FSH and LH reactive cells functioning.

### Effect on the levels of gonadotrophic hormones

The concentration of LH and FSH in blood serum at the end of the experiment. (Table 3: Blood hormone levels in the experimental group) No statistically significant differences (*p* < 0.05) were found between the groups for LH and FSH levels. The LH/FSH ratio was higher in the EV (1.79) compared to the EV + EMR group (1.35).

**Table 3:** Blood hormone levels in the experimental groups

Parameter	EV	EMR	EMR + EV	*p*-value
LH/ng/ml	4.13 ± 0.72	4.36 ± 0.63	4.21 ± 0.39	0.79
FSH/ng/ml	2.30 ± 0.10	2.68 ± 0.59	3.10 ± 0.11	0.17
LH/FSH ratio	1.79	1.62	1.35	

*p-value significant at the 95% confidence interval

## Discussion

To understand the etiopathology of PCOS, the present study used an EV-treated rat model that exhibits hormonal, reproductive, and metabolic signs similar to the PCOS condition in humans. In the present study, PCO condition was successfully induced by a single dose of 4 mg EV in young adult cyclic rats and anovulation was successfully maintained for 8 weeks as previously reported [13, 21–23].

No changes were observed in feed intake of the animals and concomitant body weight. These results were similar to a previous study conducted in the EV rat model [21].

In the present experiment, the neurons responsible for GnRH modulation were not examined. But, immunohistochemical analysis revealed LH-positive and FSH-positive staining distributed in the arcuate nucleus which is located bilaterally around the 3rd ventricle. Histological analysis of these neurons, which are grouped together in the arcuate nucleus , showed no significant structural changes in all groups. The preoptic region of the hypothalamus contains LH releasing hormone neurons that projects to the anterior pituitary via connections in the arcuate nucleus . In this study, qualitative IHC analysis revealed no significant changes in LH reactive cells. In this study, kisspeptin neurons, which are mainly responsible for GnRH stimulation, were not specifically examined. The kisspeptin neurons are specialized neurons located in the arcuate nucleus and neuroanatomically organized in the rostral periventricular area of the 3rd ventricle [24]. Approximately 50–70% of GnRH neurons express the kisspeptin receptor, so although we specifically examined the histometry of neurons in this area, we found no significant change. However, it is advisable that researchers study these neurons to understand whether the receptors (GnRHR) are active on them or not. In a previous study, electron microscopy was used to detect changes in the arcuate nucleus neurons, where an increase in the number of astrocytes was observed [21]. There are similar studies that suggest that 86 kHz EMR does not appear to have any harmful effect or cause stress on neural cells after long-term exposure [25]. While other research in the higher spectrum suggests that long-term exposure to mobile devices of 900 MHz could cause neurotoxic risk that could alter the histology of the brain and impair its function [26]. Understanding the production of GnRH, a key regulator of the HPO axis, is very useful in the treatment of PCOdue to its ability to block estrogen secretion from the ovary. The hypothalamus contains several nuclei responsible for GnRH synthesis and must all be examined carefully. The preoptic area (POA), ventromedial nucleus (VMN), arcuate nucleus, and median eminence are located around the 3rd ventricle [27, 28]. It is also important to note that when neurons are examined, no definite conclusion can be drawn since they have various functions. For example, despite their role in reproduction, kisspeptin neurons are also responsible for energy balance and metabolism. However, there is research that states that there is a link between altered energy balance and reproductive function [29], which was not examined in this research.

Secretion of GnRH neurons from the hypothalamic nuclei reaches the fenestrated capillaries of the hypothalamohypophyseal portal circulation. The primary recognition site for GnRH is the anterior pituitary where it binds to the gonadotrophin-releasing hormone receptor (GnRHR), which is a G-protein receptor present on the surface of gonadotrophs (basophilic cells [28]. The present study showed that the EV-treated group had a reduced number of basophils, a reduction in the distribution of LH reactive cells, and blood stasis. Previous reports have shown that EV induction for PCOsincreases the pituitary weight and acidophil count [21]. At the same time, it also decreases the number of basophils responsible for the production of LH and FSH [30]. This study did not show any change in the number of FSH-positive cells. In general basophils account for only 10% of the cells secreting LH and FSH in the pars distalis of the pituitary gland [31]. This gland is a homeostatic tissue and the number of acidophilic cells tends to vary with the oestrous cycle as a result of prolactin secretion. In this experiment, an increase in acidophilic cells and an irregular shape of the basophilic cells were observed in both groups with EMR exposure. Both the EMR and EMR + EV also had high positive staining for FSH and LH. Mobile EMR from previous studies showed changes in the pituitary gland and its sensitivity to EMR when exposed for 2 months and showed a decrease in LH and FSH. The same article concluded that non-ionizing radiation had shown deleterious effects on the pituitary and ovary of healthy animals [32]. Thus, assumptions can be made about a possible effect of non-ionizing EMR on healthy cells. In this current study, non-ionizing EMR confirms this conclusion and suggests that this frequency may have an effect based on the results obtained. However, there are many parameters that need to be considered, such as the time and length of exposure, the type of exposure, and the frequency of exposure.

Binding of GnRH to gonadotropin releasing hormone receptor (GnRHR) initiates downstream signalling of primary gonadotropins, FSH and LH into the bloodstream where they bind to FSH receptors and LH receptors on the granulosa and theca cells of developing follicles. In healthy animals, this would signal a number of intracellular mechanisms for follicular growth at various stages until maturity is reached. This process was observed as normal follicle development in the healthy control rats. The EV group of rats showed the development of polycystic ovaries. Immunohistochemical analysis in the EV group showed LH and FSH density to be lower than all the other groups. There were multiple dilated follicular cysts with enlarged antrum and thinner granulosa cell layers without visible oocyte. Some cysts had macrophages in the antrum along with pyknosis of granulosa cells, which was observed in immunohistochemical examination due to macrophages LH immunoreactivity. All these findings were similar to the results obtained in previous experiments performed with EV-induced rats [21, 22]. In the EMR + EV group, the development of follicles was observed at different stages. Corpora lutea and atretic follicles were also seen. Cystic follicles were smaller and less in EMR + EV group than in EV group. This can be because, this experiment showed an increase in LH and FSH reactive cells in the ovary. In the present study, 150 kHz electromagnetic radiation was observed to decrease the development of cystic follicles. In healthy animals, long-term exposure to Video Display Terminals (VDT), which are sources of IF field, showed no effect on the reproductive system [33].

Ovarian cysts resulting from EV induction may be intact, meaning that they have a large follicular antrum and innumerable granulosa cells surrounded by polyhedral thecal cells. Or in other cases, the granulosa cells are infiltrated by leukocytes, and this type is characterized by an intense process of leukocyte infiltration and desquamation of the granulosa cells. In the present study, the ovarian cysts in the EMR + EV group had a smaller antrum, thicker granulosa cell layers, and infiltration of leukocytes.

The endocrine profile of PCO induced by EV in rats showed a significant decrease in LH and FSH after 8 to 10 weeks [13, 23]. The EMR group showed an increase in the concentration levels of LH and FSH after 8 weeks of exposure. The response of LH and FSH to GnRH depends on a feedback mechanism regulated by the H-P-O axis. It is hypothesized that when these individual organs, such as the hypothalamus, secrete GnRH, it may not go directly to the target cells of the anterior pituitary, but to another unknown cell, resulting in disruption of the axis [34].

Non-ionizing radiation produces a “heating effect” on tissue from sources such as microwaves and mobile devices. This frequency does not have enough energy to cause the release of an electron from its outer shell. However, looking only at the cellular level in the pituitary and ovary, since there is no effect on the histoarchitecture of the arcuate nucleus of the hypothalamus, it is useful to look at the molecules that make up a cell and the atoms that make up a molecule. In these two organs, the cell membranes are composed of hydrogen (H), carbon (C), oxygen (O^2^), and phosphorous (P). Nerve signalling involves sodium (Na) and potassium (K). EV-induced rats have enlarged pituitary glands [35] and multiple ovarian cysts. Ovarian cysts contain fluid which also consists of these atoms (Na & P). The pituitary gland appears to function better in the EMR group, but the size was not measured in this study. Electromagentic radiation also reduced the development of cysts. It is safe to assume that these atoms play an important role since these organs are sensitive. Non-ionizing radiation is sufficient to alter the rotational, vibrational, or electronic valence configurations of atoms to produce thermal effects. This is said to have given rise to one of the postulations for the mechanism of TTF fields. It is thought to inhibit metaphase and cause metaphase arrest, prolonged mitosis, and cell death in the target tissue [36]. However, in this experiment rats were exposed, whole-body to a uniform electric field. Atoms remain stable when the forces between their nuclei are balanced. Sometimes the internal energy within an atom becomes too great and can cause atoms to become unstable. This can cause a cell to lose its structural shape. Ovarian cysts contain a fluid mixture of different ions. It can be postulated that energy can accumulate in these and cause cysts to lose their structural integrity. Cells may also be susceptible in this medium (fluid inside a cyst). When the same cells are placed in different environments, they will react differently. This means that cystic cells can be a target for EMR, compared to follicular cells in the ovary. However, the nature of our results limits us from making a complete and clear statement, as we did not examine the specific heat capacities of an ovary and cystic follicles. The energy within the cysts or in the pituitary gland was not measured. Furthermore, our experiment did not examine the target effect, which is extremely important for a study such as this and should be encouraged to be examined. There are no validated methods to study the specific heat capacity of follicular cysts, and researchers are encouraged to formulate new approaches.

Overall, the levels of LH/FSH in blood might have been improved by exposure to EMR but not significantly. This is reflective of the higher density of LH- and FSH- positive stained cells in the ovary and pituitary. Simultaneously the number of active basophilic cells in the pituitary were higher and there was more granulosa and theca cells to produce these hormones in the ovary of the EV + EMR group.

The HPO axis is easily disrupted when estrogens are induced into the body of an animal. Many studies, together with this current study showed the negative feedback that occurs at the pituitary when estrogens are taken and the deferential regulation of FSH and LH secretion that may occur depending on the dosage. The development of follicles in the ovary requires a steady feedback to the pituitary and hypothalamus. But, unfortunately, PCO results when there is a disruption in this axis. When an EMR source is added non-invasively to this axis, it does not affect the neurons surrounding the 3V of the arcuate nucleus of the hypothalamus. Still, it appears to regulate the number of LH and FSH in a developing PCO model, thus reducing the number of cystic follicles in the ovary.

A more detailed study is needed to investigate the effect of this invisible wave over 2 months in real-time. The results of this experiment suggest that the application of these fields non-invasively can be used later in the medical field once it is properly studied. It opens a new avenue for the treatment of PCOS if this is possible in the near future.

## Conclusion

This experiment concludes that 150 kHz EMR may not have any observable harmful effects on the neurons surrounding the arcuate nucleus of the hypothalamus of PCO-induced rats. But it may help to improve the number of basophilic cells and their response for LH and FSH secretion in the pituitary gland. In addition, this IF EMR field may have significantly decreased the development of follicular cysts formed by regulating and increasing the reactivity of LH and FSH receptors on theca and granulosa cells of developing follicles. The results of this experiment suggest that a similar non-invasive treatment may be possible for women with follicular cysts but requires futher investigation.

## Data Availability

All data is available for this experiment. It will not be released because there are other phases of this experiment.

## Abbreviations

C: Carbon
Catalog No.: Catalogue number
CEC: Campus Research Ethics Committee
CL: Corpora lutea
CRP: Campus Research and Publication Fund
E2: Estradiol
EMR: Electromagnetic radiation
EV: ESTRADIOL Valerate
FSH: Follicle stimulating hormone
GnRH: Gonadotropin-releasing hormone
GnRHR: Gonadotropin-releasing hormone receptor
H: Hydrogen
HPO: Hypothalamic-pituitary-ovarian
IF: Intermediate frequency
i.p.: Intraperitoneally
IRS: Immunoreactive score
K: Potassium
kHz: Kilo hertz
LH: Luteinizing hormone
n: Nano
Na: Sodium
O^2^: Oxygen
P: Phosphorous
PCO: Polycystic ovaries
PCOS: Polycystic ovarian syndrome
POA: Preoptic area
SD: Sprague Dawley
TTFs: Tumor Treating Fields
VDT: Video display terminals
VNM: Ventromedial nucleus
μ: Micro
