# Correction: Polycystic ovary rat model exposure to 150 kHz intermediate frequency: hypothalamic-pituitary-ovarian axis at the receptor, cellular, tissue, and hormone levels

**DOI:** 10.1186/s13048-023-01176-4

**Published:** 2023-05-17

**Authors:** Stephanie Mohammed, Venkatesan Sundaram, Chalapathi R. Adidam Venkata, Nikolay Zyuzikov

**Affiliations:** 1 Department of Physics, Faculty of Science and Technology, The University of the West Indies, St. Augustine, West Indies Trinidad and Tobago; 2 Department of Basic Veterinary Sciences, School of Veterinary Medicine, Faculty of Medical Sciences, The University of the West Indies, St. Augustine, West Indies Trinidad and Tobago; 3 Department of Clinical Medical Sciences, Faculty of Medical Sciences, The University of the West Indies, St. Augustine, West Indies Trinidad and Tobago

**Correction: J Ovarian Res 14, 173 (2021)**

10.1186/s13048-021-00914-w

The original article [1] contains errors in Figs. 3 and 7 which occurred during the compilation of the images into figures. Portions of the specified figures were duplicated within the article:

- Fig 3H EV = Fig 3H EMR
- Fig 6O EV = Fig 7O EV
- Fig 6O EMR+EV = Fig 7O control

**Fig. 3:**
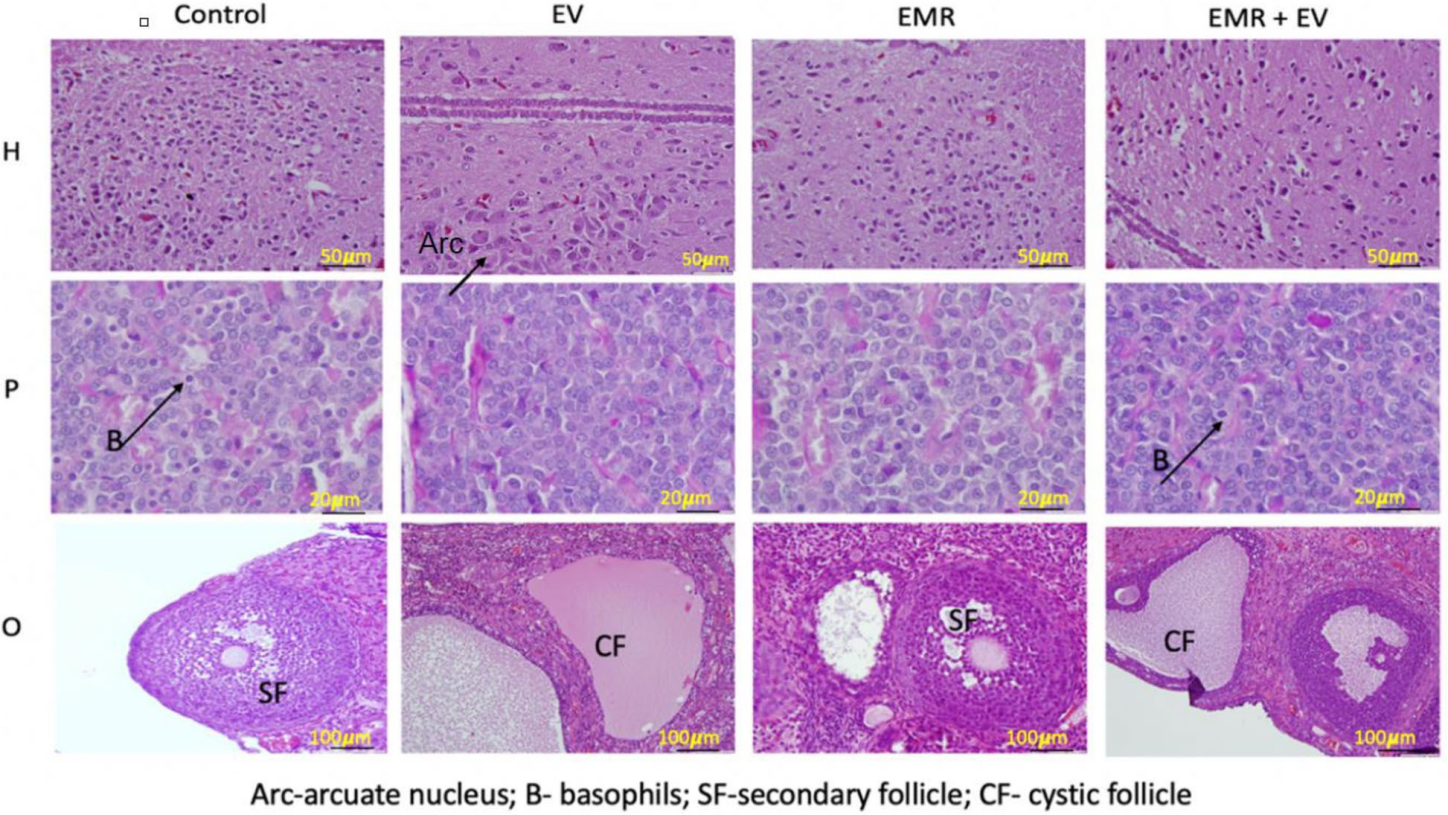
Histology of the hypothalamus, pituitary, and ovary. The photomicrograph shows neurons of the arcuate nucleus (single cluster of cells), basophil cells (poorly basophilic stained cells) of the anterior pituitary, and secondary follicle (Control-normal, EV-cystic, EMR-degenerated, EMR + EV-thin layers of granulosa, and theca cells) among each group. Arc-arcuate nucleus; B-basophil cell; SF-secondary follicle; CF – cystic follicle

**Fig. 7:**
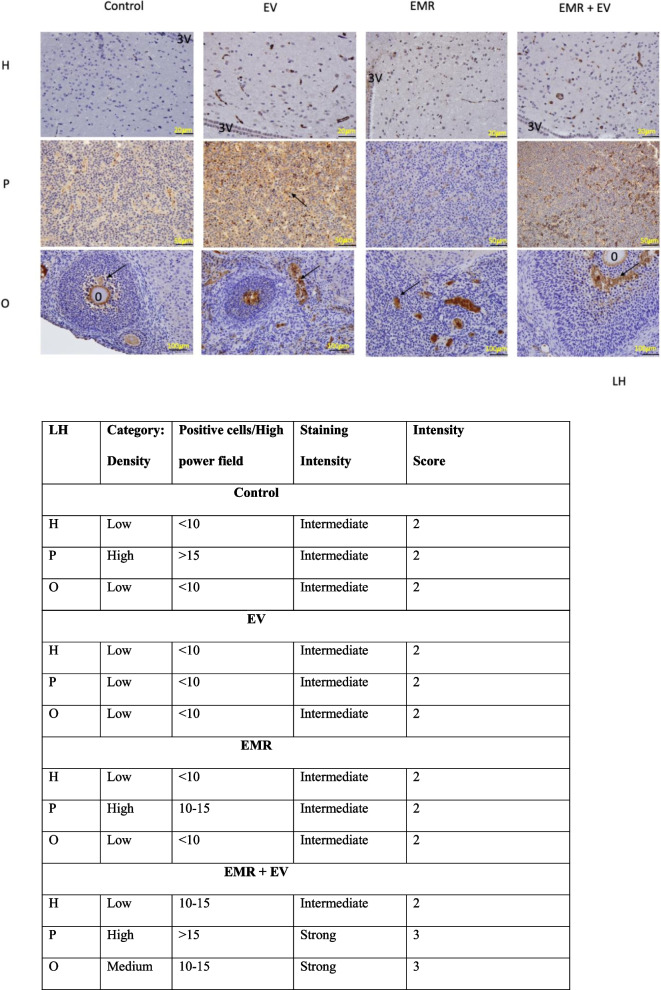
Immunohistochemical analysis of LH reactive cells of the HPO axis. Photomicrograph showing the neurons in the arcuate nucleus around the third ventricle (3 V) in the hypothalamus (H), LH reactive cells in the pituitary (P), and matured follicles with oocyte (o) and a comparison of the theca and granulosa cell layers among each group. Simultaneously there is a table showing qualitative analysis for FSH reactive cells (brown color with arrow pointing) among the groups in the HPO axis. Table showing modified Allred scoring method

Figures 3 and 7 have been corrected and shown in the following pages of this article

The authors sincerely apologize for the errors. The errors do not affect the conclusion of the article.

## References

[1] Mohammed S., Sundaram V., Adidam Venkata C.R (2021). Polycystic ovary rat model exposure to 150 kHz intermediate frequency: hypothalamic-pituitary-ovarian axis at the receptor, cellular, tissue, and hormone levels. J Ovarian Res.

